# The Skeletal Oncology Research Group Machine Learning Algorithm (SORG-MLA) for predicting prolonged postoperative opioid prescription after total knee arthroplasty: an international validation study using 3,495 patients from a Taiwanese cohort

**DOI:** 10.1186/s12891-023-06667-5

**Published:** 2023-07-05

**Authors:** Cheng-Chen Tsai, Chuan-Ching Huang, Ching-Wei Lin, Paul T. Ogink, Chih-Chi Su, Shin-Fu Chen, Mao-Hsu Yen, Jorrit-Jan Verlaan, Joseph H. Schwab, Chen-Ti Wang, Olivier Q. Groot, Ming-Hsiao Hu, Hongsen Chiang

**Affiliations:** 1 Department of Medical Education, National Taiwan University Hospital, Taipei, Taiwan; 2 Department of Orthopaedic Surgery, National Taiwan University Hospital, No.7 Chung-Shan South Road, Taipei, 10002 Taiwan; 3 Department of Orthopedic Surgery, Chang Gung Memorial Hospital, Taoyuan, Taiwan; 4 Department of Medical Education, Chang Gung Memorial Hospital, Taoyuan, Taiwan; 5 Department of Orthopaedics, University Medical Center Utrecht, Utrecht, The Netherlands; 6 Department of Computer Science and Engineering, National Taiwan Ocean University, Taipei, Taiwan; 7 Department of Orthopaedic Surgery, Massachusetts General Hospital, Boston, USA; 8 Department of Biomedical Engineering, College of Medicine and College of Engineering, National Taiwan University, Taipei, Taiwan

**Keywords:** Prolonged opioid use, Total knee arthroplasty, Machine learning, Prediction model, Asian group, Acetaminophen use

## Abstract

**Background:**

Preoperative prediction of prolonged postoperative opioid use (PPOU) after total knee arthroplasty (TKA) could identify high-risk patients for increased surveillance. The Skeletal Oncology Research Group machine learning algorithm (SORG-MLA) has been tested internally while lacking external support to assess its generalizability. The aims of this study were to externally validate this algorithm in an Asian cohort and to identify other potential independent factors for PPOU.

**Methods:**

In a tertiary center in Taiwan, 3,495 patients receiving TKA from 2010–2018 were included. Baseline characteristics were compared between the external validation cohort and the original developmental cohorts. Discrimination (area under receiver operating characteristic curve [AUROC] and precision-recall curve [AUPRC]), calibration, overall performance (Brier score), and decision curve analysis (DCA) were applied to assess the model performance. A multivariable logistic regression was used to evaluate other potential prognostic factors.

**Results:**

There were notable differences in baseline characteristics between the validation and the development cohort. Despite these variations, the SORG-MLA (https://sorg-apps.shinyapps.io/tjaopioid/) remained its good discriminatory ability (AUROC, 0.75; AUPRC, 0.34) and good overall performance (Brier score, 0.029; null model Brier score, 0.032). The algorithm could bring clinical benefit in DCA while somewhat overestimating the probability of prolonged opioid use. Preoperative acetaminophen use was an independent factor to predict PPOU (odds ratio, 2.05).

**Conclusions:**

The SORG-MLA retained its discriminatory ability and good overall performance despite the different pharmaceutical regulations. The algorithm could be used to identify high-risk patients and tailor personalized prevention policy.

**Supplementary Information:**

The online version contains supplementary material available at 10.1186/s12891-023-06667-5.

## Introduction

Total knee arthroplasty (TKA) is the definitive treatment for end-stage osteoarthritis [1], and the number of patients undergoing TKA is expected to increase substantially with the growing elderly population. In the United States, an estimated 14 million individuals aged 25 years and above manifest symptomatic knee osteoarthritis, and more than half of those diagnosed will undergo initial TKA surgery prior to their demise, accounting for over 600,000 TKAs conducted annually [2]. Although the satisfaction rate of TKA is relatively high [2], 60% of the patients suffer from severe postoperative knee pain after TKA and 30% of them described such pain as moderate [3]. This discomfort negatively affects early postoperative ambulation and rehabilitation; therefore, analgesics are commonly used for the patients’ better recovery. Patients who undergo TKA surgery are typically prescribed a two to three-week course of non-steroid anti-inflammation drugs (NSAIDs) as a conventional treatment for pain relief [4, 5]. However, chronic postoperative pain affects about 20% of TKA recipients, and NSAIDs provide an average pain reduction of approximately 25%. Moreover, individuals with contraindications like allergic reactions, treatment-resistant hypertension, increased cardiovascular disease risk, and severe chronic kidney disease may not be suitable candidates for NSAID treatment [5]. Therefore, opioids have been used as an important part of multimodal postoperative analgesic regimen. Bedard et al. posited that roughly one-third of patients scheduled for total knee arthroplasty (TKA) had utilized opioids in the three-month period leading up to the surgical intervention [6]. However, their use is unfavorably associated with postoperative comorbidities, such as prolonged hospitalization, greater costs, and increased readmissions [7]. Therefore, a customized opioid prescription for these patients would be valuable.

Several risk factors for prolonged postoperative opioid use (PPOU) have already been identified, including preoperative opioid use, female gender, patients aged < 50 years, greater length of stay, and worse health status [6], however, amongst them are complex interactions. The machine learning algorithm offers flexible estimations and would help to establish a preoperative prediction of opioid use after the surgery [8, 9]. Katakam et al. developed the Skeletal Oncology Research Group machine learning algorithm (SORG-MLA) to predict PPOU after TKA in the United States. The algorithm was trained on a dataset of 12,542 institutional patients, and was successfully tested in the internal validation [8]. However, international variations about medical philosophies and pharmacy-restrictions would bias the abroad application of this algorithm [9]. An external validation in a geographically distant cohort with significant medicolegal and cultural differences is thus necessary.

The American Academy of Orthopaedic Surgeons (AAOS) recommended the preoperative use of acetaminophen in 2021 [10]. Acetaminophen provides pain relief for patients with a lower risk of acute kidney injury or gastrointestinal side effects compared to NSAIDs. However, there have been reports associating the preoperative use of acetaminophen with postoperative opioid use (PPOU) [11]. The developmental study of SORG-MLA, which did not incorporate the preoperative use of acetaminophen, was possibly conducted before the AAOS recommendation for this medication. It would be interesting to investigate whether the performance of SORG-MLA could be improved by including acetaminophen as a potential prediction factor.

Together, this study aimed to answer the following two questions. (1) Was the SORG-MLA for predicting PPOU after TKA generalizable, measured by area under the precision-recall curve (AUPRC) as well as other performance metrics, to a geographically, socioeconomically distinct cohort? (2) Is preoperative acetaminophen use an independent factor associated with PPOU while controlling for the SORG-MLA predictions?

## Material and methods

### Guideline

This retrospective validation study was performed under the guidance of the Transparent Reporting of a Multivariable Prediction Model for Individual Prognosis or Diagnosis (TRIPOD) statement [12]. The study was approved by the Research Ethics Committee of the authors’ hospital (Case Number 202106028RINA), and written informed consent was obtained from all subjects.

### Study design

**Fig. 1:**
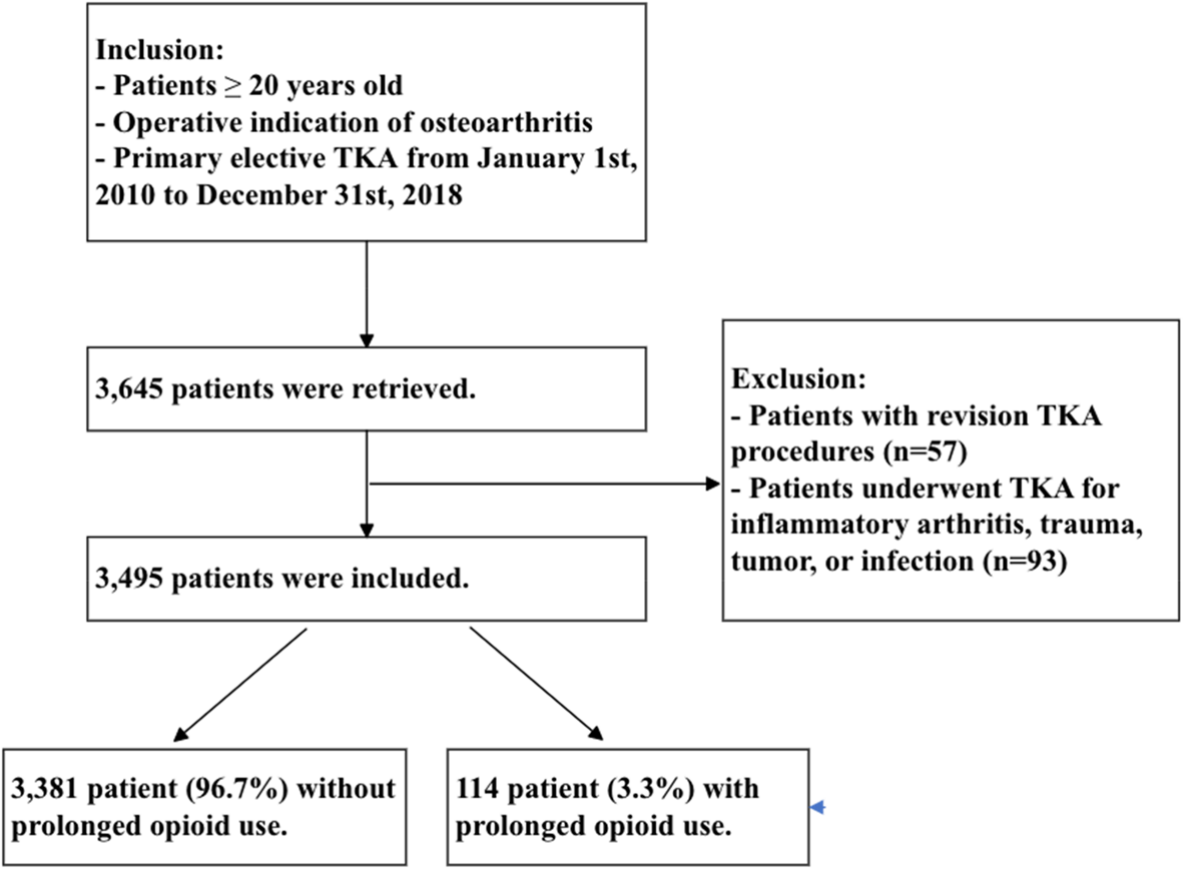
Study flowchart showing the enrolled patients in the validation cohort. TKA, total knee arthroplasty

A retrospective study was conducted at this single tertiary health care system in Taiwan. Inclusion criteria for the study-subjects were: (1) age 20 years or older, (2) indicated for operation of knee osteoarthritis, and (3) receiving primary elective TKA during January 1^st^ 2010 to December 31^st^ 2018. Exclusion criteria were those received: (1) revision TKA and (2) TKA performed for inflammatory arthritis, trauma, tumor, or infection (Fig. 1). The same criteria were used as the development cohort except for age, because the patients aged 18 to 20 years were excluded by the Research Ethics Committee’s regulation in this incidence due to that they are legally minor. Such exclusion has not affected our final analysis because knee osteoarthritis is primarily degenerative in nature and less than 0.1% of our patients were younger than 30-year-old at the time of the surgery.

### Outcome

PPOU were defined as the presence of continuous opioid prescriptions within three specific time intervals following the index surgery: first 30 days, 30–90 days, and 90–180 days after surgery [13]. The complete list of opioid medications is referred to the development study [8]. The complete list of opioid medications is referred to the development study [8].

### Development cohort

In the developmental study, 12,542 patients underwent TKA for osteoarthritis and 1125 (9.0%) received prolonged postoperative opioid prescriptions. The median age was 67 years (interquartile range 60–74) and 7559 (60.3%) patients were female. In the year before surgery, 2631 (21.0%) patients received opioid prescription. The other most prescribed medicines preoperatively were NSAIDs (*n* = 2164, 17.3%), beta-blocker (*n* = 1742, 13.9%), and immunosuppressant (*n* = 1431, 11.4%). The most prevalent comorbidities were arrhythmias (*n* = 1506, 12.0%), followed by diabetes (*n* = 1182, 9.4%), and chronic obstructive pulmonary disease (*n* = 936, 7.5%) [8].

### Data collection and predictors

The following variables were manually retrieved from the medical records: patient demographics (age, sex, marital status, ethnicity), disposition (inpatient or outpatient), preoperative laboratory values (white blood cell count, hemoglobin, platelet count, creatinine, alanine transaminase [ALT]), neighborhood characteristics from Taiwan National Department of Household Registration online database based on patient’s living area zip code (median household income, educational level, median age, neighborhood unemployment rate, population density), preoperative medications (angiotensin-converting enzyme inhibitor, acetaminophen, angiotensin II receptor blocker, anti-depressants, beta-2 agonists, beta-blockers, benzodiazepines, gabapentin and pregabalin, immunosuppressants, nonsteroidal anti-inflammatory drugs, opioid, anti-psychotics), and preoperative comorbidities (tobacco use, alcohol abuse, drug abuse, diabetes, renal failure, depression, psychoses, myocardial infarction, congestive heart failure, peripheral vascular disease, cerebrovascular accidents, chronic obstructive pulmonary disease, arrhythmias, valvular disease, liver disease, malignancy). The same definitions for outcome and predictor variables were used as the original development study [8]. The authors of the development study were not part of the data extraction or analysis. The same definitions for outcome and predictor variables were used as the original development study [8]. The authors of the development study were not part of the data extraction or analysis.

**Fig. 2:**
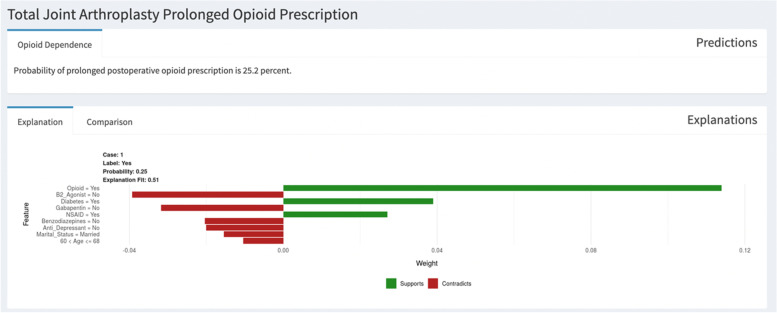
Patient-specific explanation for prediction generated by the online SORG-MLA model at https://sorg-apps.shinyapps.io/tjaopioid/

The SORG-MLA used nine variables to provide a prediction of PPOU: age, history of preoperative opioid use, marital status, diagnosis of diabetes, and preoperative medications (antidepressants, benzodiazepines, nonsteroidal anti-inflammatory drugs, gabapentin, and beta-2 agonists). An example of an individual patient-level explanation for the model predictions is shown in Fig. 2. The predicted probability of PPOU was 0.25 for a 65-year-old patient with diabetes and a history of opioid use and NSAID use. We provided an easy step-by-step guide, using a dummy dataset, for validation of open accessible prediction models (Additional file 1). This flow-chart as well as the open-source code may help the other studies to implement the external validation of prediction models.

### Missing data

The proportions of missing data were all less than 30% [14], and the MissForest algorithm was applied to impute the missing data for the following variables: unemployment rate in 25 patients (0.7%); neighborhood characteristics in 7 patients (0.2%); creatinine in 4 patients (0.11%); hemoglobin in 2 patients (0.06%); white blood cell in 1 patient (0.03%); and platelet in 1 patient (0.03%). The outcome of interest could be retrieved through the database of the National Health Insurance registry and no opioid drugs could be obtained without prescriptions; therefore, the missing proportion of PPOU was regarded as none. The proportions of missing data were all less than 30% [14], and the MissForest algorithm was applied to impute the missing data for the following variables: unemployment rate in 25 patients (0.7%); neighborhood characteristics in 7 patients (0.2%); creatinine in 4 patients (0.11%); hemoglobin in 2 patients (0.06%); white blood cell in 1 patient (0.03%); and platelet in 1 patient (0.03%). The outcome of interest could be retrieved through the database of the National Health Insurance registry and no opioid drugs could be obtained without prescriptions; therefore, the missing proportion of PPOU was regarded as none.

A sensitivity test was conducted by using only individuals who have no missing data, and the result was similar to the one with imputation of missing data.

### Participants’ baseline characteristics

In total, 3,495 patients were included in this study and 114 (3.3%) among them received PPOU. 2,687 (77%) patients were female with a median age of 73 (interquartile range [IQR], 67 to 78) years. The most prescribed medicines preoperatively were NSAIDs (*n* = 3,453, 99%), followed by ARBs (*n* = 779, 22%) and beta-blockers (*n* = 607, 17%). The patients in the developmental and the validation cohorts differed in many regards. Generally, patients in the validation cohort were older, married more often, earned less money, had more inpatient dispositions, had less depression or drug abuse as the comorbidities, and had a more common pharmaceutical history of taking NSAIDs, angiotensin receptor blocker, beta-blockers, benzodiazepines, gabapentin, and anti-psychotics (Table 1). Slightly more than one-tenth of the patients (*n* = 422, 12%) had been prescribed with acetaminophen. The comparison of baseline characteristics between patients with and without PPOU is presented in Table 2. Additionally, all patients in the validation cohort were covered by the National Health Insurance whereas the patients in developmental cohorts in the US usually pertained to various insurances.

**Table 1:** Comparison of baseline characteristics between the development cohort (*n* = 12,542) and the external validation cohort (*n* = 3,495)

Variable	**n (%) | median (IQR)**		***p*** **-value**
	Development cohort (*n* = 12,542)	Validation cohort (*n* = 3,495)	
Age (years)	67.0 (60.0–74.0)	73.0 (67.0–78.0)	< 0.001
Female sex	7,559 (60.3)	2,687 (76.9)	< 0.001
Race			
Non-white	1,214 (10.0)	3,495 (100.0)	
White	10,947 (90.0)	-	
Ethnicity			
Hispanic	268 (2.2)	-	
Non-Hispanic	11,893 (97.8)	3,495 (100.0)	
Married	7,575 (62.6)	2,526 (72.3)	< 0.001
Veteran	1,597 (13.6)	-	
Disposition			
Inpatient	11,683 (93.2)	3,495 (100.0)	
Outpatient	859 (6.8)	-	
Laboratory values			
Hemoglobin (g/dl)	13.6 (12.7–14.5)	13.0 (12.0–13.9)	< 0.001
White blood cell (× 10^3^/µL)	6.90 (5.80–8.20)	6.87 (5.72–8.46)	0.520
Platelet (× 10^3^/µL)	254.0 (213.0–301.0)	220.0 (183.0–262.0)	< 0.001
Creatinine (mg/dL)	0.90 (0.76–1.05)	0.8 (0.7–1.0)	< 0.001
Insurance			
Medicaid	529 (4.2)	-	
Medicare	6,469 (51.6)	-	
Neighborhood characteristics			
Median household income (USD)	80,375 (62,114–100,674)	21,233 (19,666–23,433)	< 0.001
Age (years)	41.9 (37.6–44.8)	43.0 (41.0–44.0)	< 0.001
High school education (%)	24 (15–30)	76.8 (73.1–84.7)	< 0.001
Unemployment rate (%)	5.7 (4.6–7.3)	3.8 (3.8–3.8)	< 0.001
Medications			
ACEi	1,366 (10.9)	106 (3.0)	< 0.001
Acetaminophen	-	422 (12.1)	-
ARB	655 (5.2)	779 (22.3)	< 0.001
Anti-depressants	1,191 (9.5)	251 (7.2)	< 0.001
Beta-2-agonist	833 (6.6)	47 (1.3)	< 0.001
Beta-blocker	1,742 (13.9)	607 (17.4)	< 0.001
Benzodiazepines	992 (7.9)	530 (15.2)	< 0.001
Gabapentin	512 (4.1)	251 (7.2)	< 0.001
Immunosuppressant	1,431 (11.4)	43 (1.2)	< 0.001
NSAID	2,164 (17.3)	3,453 (98.8)	< 0.001
Opioid	2,631 (21.0)	360 (10.3)	< 0.001
Anti-psychotic	205 (1.6)	513 (14.7)	< 0.001
Comorbidities			
Tobacco use	174 (1.4)	172 (4.9)	< 0.001
Drug abuse	76 (0.6)	6 (0.2)	0.001
Diabetes	1,182 (9.4)	602 (17.2)	< 0.001
Renal failure	346 (2.8)	189 (5.4)	< 0.001
Depression	803 (6.4)	157 (4.5)	< 0.001
Psychoses	73 (0.6)	11 (0.3)	0.050
Myocardial infarction	298 (2.4)	492 (14.0)	< 0.001
Congestive heart failure	470 (3.7)	176 (5.0)	< 0.001
Peripheral vascular disease	474 (3.8)	185 (5.3)	< 0.001
Cerebrovascular accident	407 (3.2)	349 (10.0)	< 0.001
COPD	936 (7.5)	342 (9.8)	< 0.001
Arrhythmias	1,506 (12.0)	262 (7.5)	< 0.001
Valvular disease	612 (4.9)	105 (3.0)	< 0.001
Liver disease	265 (2.1)	221 (6.3)	< 0.001
Malignancy	551 (4.4)	669 (19.1)	< 0.001
Prolonged opioid use	1,125 (9.0)	114 (3.3)	< 0.001

*IQR* Interquartile range; *USD* United States Dollar; *NIH* National Health Insurance; *ACEi* Angiotensin converting enzyme inhibitors; *ARB* Angiotensin receptor blockers; *NSAID* Non-steroidal anti-inflammatory drugs; *COPD* Chronic obstructive pulmonary disease

**Table 2:** Comparison of baseline characteristics between patients with and without prolonged postoperative opioid used

Variable	**n (%) | median (IQR)**		***p*** **-value**
	Prolonged postoperative opioid used (*n* = 114)	Non-prolonged postoperative opioid used (*n* = 3,381)	
Age (years)	72.0 (64.3–78.0)	73.0 (67.0–78.0)	0.322
Female sex	93 (81.6)	2594 (76.7)	0.226
Married	81 (71.1)	2445 (72.3)	0.767
Laboratory values			
Hemoglobin (g/dl)	12.9 (12.0–14.1)	13.0 (12.0–13.9)	0.396
White blood cell (× 10^3^/µL)	7.2 (5.9–9.2)	6.9 (5.7–8.4)	0.150
Platelet (× 10^3^/µL)	219.0 (182.3–259.8)	220.0 (183.0–262.0)	0.927
Creatinine (mg/dL)	0.8 (0.7–1.1)	0.8 (0.7–1.0)	0.491
Neighborhood characteristics			
Median household income (USD)	21.0 (19.4–23.3)	21.3 (19.7–23.4)	0.651
Age (years)	43.0 (41.0–44.0)	43.0 (41.0–44.0)	0.455
High school education (%)	76.2 (71.4–84.7)	77.2 (73.1–84.7)	0.166
Unemployment rate (%)	3.8 (3.8–3.8)	3.8 (3.8–3.8)	0.722
Medications			
ACEi	3 (2.6)	44 (1.3)	0.225
Acetaminophen	23 (20.2)	399 (11.8)	**0.007**
ARB	22 (19.3)	584 (17.3)	0.574
Anti-depressants	14 (12.2)	169 (5.0)	** < 0.001**
Beta-2-agonist	3 (2.6)	22 (0.6)	**0.014**
Beta-blocker	19 (1.7)	456 (13.5)	0.330
Benzodiazepines	25 (21.9)	505 (14.9)	**0.041**
Gabapentin	14 (12.3)	237 (7.0)	**0.032**
Immunosuppressant	7 (6.1)	35 (1.0)	** < 0.001**
NSAID	76 (66.7)	2050 (60.6)	0.194
Opioid	47 (41.2)	313 (9.3)	** < 0.001**
Anti-psychotic	4 (3.5)	52 (1.5)	0.099
Comorbidities			
Tobacco use	6 (5.3)	166 (4.9)	0.766
Drug abuse	0 (0.0)	6 (0.2)	0.653
Diabetes	29 (25.4)	573 (16.9)	**0.018**
Renal failure	11 (9.6)	178 (5.3)	**0.042**
Depression	13 (11.4)	144 (4.3)	** < 0.001**
Psychoses	0 (0.0)	11 (0.3)	0.542
Myocardial infarction	23 (20.2)	469 (13.9)	0.057
Congestive heart failure	12 (10.5)	164 (4.9)	**0.006**
Peripheral vascular disease	12 (10.5)	173 (5.1)	**0.011**
Cerebrovascular accident	18 (15.8)	331 (9.8)	**0.036**
COPD	24 (21.1)	318 (9.4)	** < 0.001**
Arrhythmias	10 (8.8)	252 (7.5)	0.599
Valvular disease	5 (4.4)	100 (3.0)	0.380
Liver disease	11 (9.6)	210 (6.2)	0.138
Malignancy	33 (28.9)	636 (18.8)	**0.007**

*IQR* Interquartile range; *USD* United States Dollar; *NIH* National Health Insurance; *ACEi* Angiotensin converting enzyme inhibitors; *ARB* Angiotensin receptor blockers; *NSAID* Non-steroidal anti-inflammatory drugs; *COPD* Chronic obstructive pulmonary disease

**Bold**, *p*-value < 0.05

### Statistical analysis

All predictions were retrieved from the online application at https://sorg-apps.shinyapps.io/tjaopioid/. Baseline characteristics were compared between the two cohorts: one representing the validation cohort in Taiwan, and another representing the development cohort from Katakam et al. in the US. For continuous variables, one-way median tests were conducted, while the chi-square tests were performed for categorical variables. The discrimination as being measured by the area under the receiver characteristic curve (AUROC) and the area under the precision-recall curve (AUPRC), the calibration, the Brier score for overall performance, and the decision curve analysis were applied to evaluate the performance of SORG-MLA. The detailed explanation of the applied performance methods was provided in Additional file 1 [15]. The detailed explanation of the applied performance methods was provided in Additional file 1 [15]. We performed a multivariable logistic regression analysis to incorporate the serum ALT level into the model of SORG-MLA while examining the preoperative use of acetaminophen as a predictor for PPOU. The rationale behind incorporating serum ALT level in the analysis was that some patients with compromised liver function were not prescribed acetaminophen due to concerns about its hepatotoxicity, particularly considering the high prevalence of hepatitis B and C in Taiwan [16, 17]. The multivariable logistic regression results are presented as odds ratios (ORs) with 95% confidence intervals (CIs).

## Results

### Was the SORG-MLA for predicting PPOU after TKA generalizable, measured by AUPRC as well as other performance metrics, to a geographically, socioeconomically distinct cohort?

**Fig. 3:**
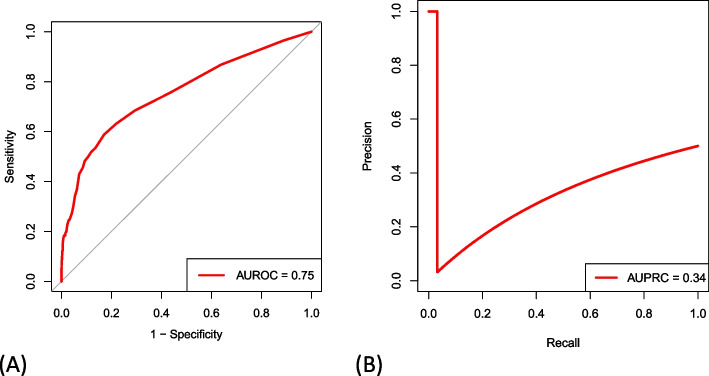
Area under **A** the receiver operating characteristic curve (AUROC) and **B** precision-recall curve (AUPRC) for SORG-MLA model

**Fig. 4:**
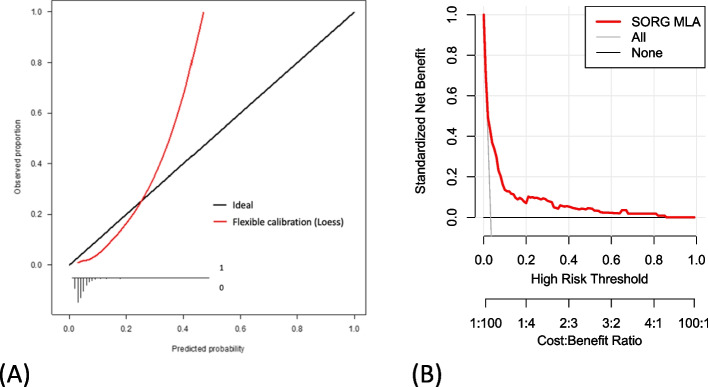
**A** Calibration plot and **B** decision curve analysis with standardized net benefit by threshold probability for SORG-MLA model

The algorithm achieved an AUROC of 0.75 (95% CI, 0.70 to 0.81; Fig. 3A and Table 3) and AUPRC of 0.34 (95% CI, 0.34 to 0.52; Fig. 3B) on external validation. It overestimated the observed proportion of patients with sustained opioid prescription, as being shown by the negative calibration intercept of -0.82 (95% CI, -1.01 to -0.63; Fig. 4A) with a calibration slope of 1.49 (95% CI, 1.25 to 1.73). The actual rate of PPOU was significantly lower than the rate predicted by SORG-MLA (3.3% versus 6.9%; one-sample t-test, *p* < 0.05). The raw Brier score was 0.029 compared to the score of 0.032 in the null model. The decision curve representing the SORG-MLA was, in a wide range of threshold probabilities, above the other two curves representing two default strategies of changing managements did, either for all patients or for no patients (Fig. 4B). These results indicate the algorithm could bring net benefit, compared to the two strategies, in a wide variety of clinical scenarios.

**Table 3:** Performance metrics of development cohort and external validation cohort

Metrics	Development cohort (*n* = 12,542)	Validation cohort (*n* = 3,495)
Discrimination		
AUROC	0.76	0.75 (0.70 to 0.81)
AUPRC	Not available	0.34 (0.34 to 0.52)
Calibration		
Intercept	0.16	-0.82 (-1.01 to -0.63)
Slope	1.08	1.49 (1.25 to 1.73)
Brier score	0.073 (0.082)	0.029 (0.032)

The 95% confidence intervals or null model Brier scores are provided between parentheses

*AUROC* Area under the receiver operating characteristic; *AUPRC* Area under the precision-recall curve

### Is preoperative acetaminophen use an independent factor associated with PPOU while controlling for the SORG-MLA predictions?

Among 422 patients taking acetaminophen preoperatively, 23 were found to have prolonged postoperative opioid use, while 91 out of 3,073 patients not taking acetaminophen had PPOU. In univariate analysis, patients taking acetaminophen preoperatively tended to depend on a more prolonged use of opioids for postoperative pain (OR, 1.89; 95% CI, 1.16 to 2.97). In the multivariable analysis, which adjusted for SORG-MLA predictions and the patient’s serum ALT level, such trend remained significant (OR, 2.05; 95% CI, 1.03 to 3.82).

## Discussion

An accurate prediction of the risk of PPOU could facilitate clinical decisions to avoid excessive use of opioids and the subsequent adverse events. The SORG-MLA was developed to predict patient-specific risks on PPOU after TKA [8]. Although the algorithm has been internally validated, its generality remains questionable and requires the validations with diverse, external cohorts according to the suggestion in the TRIPOD guidelines. The SORG-MLA was developed to predict patient-specific risks on PPOU after TKA [8]. Although the algorithm has been internally validated, its generality remains questionable and requires validations with diverse, external cohorts according to the suggestion in the TRIPOD guidelines [12]. In this study, we validated the algorithm with a geographically, socioeconomically distinct cohort and found it held good discriminatory ability and overall performance, except the model had a tendency to overestimate the probability of PPOU. We also reported an AUPRC of 0.34, indicating a minimal concern about the excessive optimism on the algorithm’s discriminatory ability [18]. Also, the model could be improved upon by incorporating preoperative acetaminophen use as a risk factor.

### Limitations

There are several limitations in this study. First, this was a single-center retrospective study that might not be adequately representative to other global districts or human subjects [19–21]. Some potential selection biases could not be avoided. Second, the time-period of this study (2010–2018) was different from that of the developmental cohort (2000–2018). Because both the physicians and the patients have recently become more alert to the PPOU [22], the historical background between the two cohorts might be different. Third, there are major differences in pharmaceutical regulation and prescription philosophy between the two countries [23]. Despite the historical and legislative differences, the algorithm has held its good discriminatory ability and overall performance as being successfully tested by decision curve analysis in our geographically distinct cohort [22, 22]. The difference in PPOU between both cohorts could be influenced by various factors including pharmaceutical philosophy, legal regulations, and socioeconomic factors. Therefore, the importance of an external validation study with a distance cohort could not be over-emphasized. Opioid prescriptions may vary internationally and even domestically [24, 25]. Opioid prescriptions may vary internationally and even domestically [24, 25]. For instance, the prescription of opioids in Taiwan is strictly regulated by the local Food and Drug Administration. The prescriptions for strong oral opioids, such as morphine and oxycodone, are strongly discouraged for non-cancer patients. The physicians who prescribe long term opioids are mandated to submit clinical report-forms quarterly. These country-wise differences may lead to different patterns of analgesics-usage. Almost all patients in our validation cohort were prescribed with NSAIDs as the first-line non-surgical treatments, compared with 17% in the developmental cohort. Furthermore, only 10% patients in our cohort had received preoperative opioids, whereas twice as many (21%) patients in the US cohort had done so. The preoperative use of opioids and NSAIDs have been determined as the 1^st^ and the 6^th^ most important factors, respectively, to predict PPOU after TKA [8]. Remarkably, despite of these distinct differences in the two important variables at baseline, the SORG-MLA was still capable of maintaining good discrimination and overall performance [8]. Inflammation is a closely integrated processes in knee osteoarthritis and may affect disease progression and pain [26]. Several cytokines, such as prostaglandin E2, played indispensable roles in the degeneration and inflammation course [27]. Therefore, the use of oral NSAIDs, an anti-inflammation and an analgesic agents work by inhibiting the activity of cyclooxygenase enzymes, were recommended in the 2010s for patients with knee osteoarthritis [28]. Inflammation are closely integrated processes in knee osteoarthritis and may affect disease progression and pain [26]. Several cytokines, such as prostaglandin E2, played indispensable roles in the degeneration and inflammation course [27]. Therefore, the use of oral NSAIDs, an anti-inflammation and analgesic agents work by inhibiting the activity of cyclooxygenase enzymes, was recommended in the 2010s for patients with knee osteoarthritis [28]. In contrast, the use of acetaminophen was inconclusively recommended by the AAOS in that time. This recommendation was not affirmed until 2021 by its third edition treatment guideline, possibly since acetaminophen was less efficacious than NSAIDs. We found NSAIDs to be the most commonly used medication in our validation-cohort and the second most used in the developmental cohort, possibly because of following these recommendations. In contrast, the European Society for Clinical and Economic Aspects of Osteoporosis, Osteoarthritis and Musculoskeletal Diseases (ESCEO) has recommended oral NSAIDs only if acetaminophen or topical NSAIDs provide unsatisfactory relief of symptoms [29]. The discrepancy between the two academies might indicate a different pharmaceutical philosophy across the Atlantic Ocean. The authors of the developmental study have practiced in the US and may have ignored the preoperative use of acetaminophen by their patients as a risk factor. On the other hand, such risk has been identified in some previous studies [11], and the postoperative use of acetaminophen has been considered a protective factor [30]. The geographic discrepancy should also highlight the importance of an external validation study to test the generalizability in this case [31]. The geographic discrepancy should also highlight the importance of an external validation study to test the generalizability in this case [31].

In this study, we demonstrated that the preoperative use of acetaminophen was an independent risk factor while controlling for the SORG-MLA prediction and the patients’ serum ALT level. Considering the notorious hepatotoxicity of acetaminophen [32], we felt obligated to include the serum ALT level, serving as a surrogate of hepatic function, in our multivariable analysis to provide a more reliable prognostic estimation. Considering the notorious hepatotoxicity of acetaminophen [32], we felt obligated to include the serum ALT level, serving as a surrogate of hepatic function, in our multivariable analysis to provide a more reliable prognostic estimation. The results suggested that the SORG-MLA could be further improved by considering the incremental factor. Unfortunately, in this study, we failed to retrain the SORG-MLA to provide a more comprehensive evaluation by adding the medication use as a new prognostic factor. In order to avoid over-fitting and to provide a more generalizable prediction, we should retrain the model with both the American and Taiwanese cohorts. However, for security and ethical reasons, sharing such big data from two countries mandated complicated processes and considerable time as long as several months for application. Therefore, we finally decided to report our preliminary findings and hoped the results could inspire more related studies. Future research are needed to investigate the effects of additional predictive factors such as the uses of pharmaceutical grade glucosamine and chondroitin sulfate, and to develop or retrain a prediction model to provide more personalized prediction [29].

## Conclusions

Despite different baseline characteristics and pharmaceutical regulations, the SORG-MLA for PPOU after TKA held good discriminative abilities and good overall performance in a geographically distinct region. The physicians in Taiwan could adopt this algorithm to identify high-risk patients before TKA surgeries and to tailor individualized preventive strategies for postoperative pain-control. The trend between preoperative acetaminophen uses and PPOU indicates that acetaminophen may be a risk factor for an extended postoperative opioid use. It also highlights the importance of investigating additional factors to further improve prediction models to get more personalized PPOU prediction.

## Supplementary Information

**Additional file1.**

## Data Availability

The datasets used and analyzed during the current study available from the corresponding author on reasonable request.

## Abbreviations

PPOU: Prolonged postoperative opioid use
TKA: Total knee arthroplasty
SORG-MLA: Skeletal Oncology Research Group machine learning algorithm
AUROC: Area under receiver operating characteristic curve
AUPRC: Area under precision-recall curve
DCA: Decision curve analysis
AAOS: Academy of Orthopaedic Surgeons
NSAIDS: Non-steroid anti-inflammation drugs
TRIPOD: Transparent Reporting of a Multivariable Prediction Model for Individual Prognosis or Diagnosis
ALT: Alanine transaminase
ORs: Odds ratios
CIs: Confidence intervals
IQR: Interquartile range
ESCEO: European Society for Clinical and Economic Aspects of Osteoporosis, Osteoarthritis and Musculoskeletal Diseases
