# Supplementary material for: The Skeletal Oncology Research Group Machine Learning Algorithm (SORG-MLA) for predicting prolonged postoperative opioid prescription after total knee arthroplasty: an international validation study using 3,495 patients from a Taiwanese cohort
Source: BMC Musculoskelet Disord. 2023 Jul 5;24:553. doi: 10.1186/s12891-023-06667-5 (PMC10320986; doi:10.1186/s12891-023-06667-5)
Supplement: Supplementary file 1 — Additional file1. [file 12891_2023_6667_MOESM1_ESM.docx]

**Appendix 1.** Step by step guide for validation of the open accessible prediction model for total knee arthroplasty.

**Step 1.**

- Open your own Google drive.
- Create a folder named “SORG-opioid-TKR”
- Create a comma-separated values (CSV) file named “Data_and_results_for_SORG_opioid_TKR”
- Create a Google Colaboratory named “SORG-opioid-TKR web-crawler.ipynb”

*What if, you could not find a Google Collaboratory?*

- You can click the right mouse button, choose “More”, and then choose “Connect more apps”.

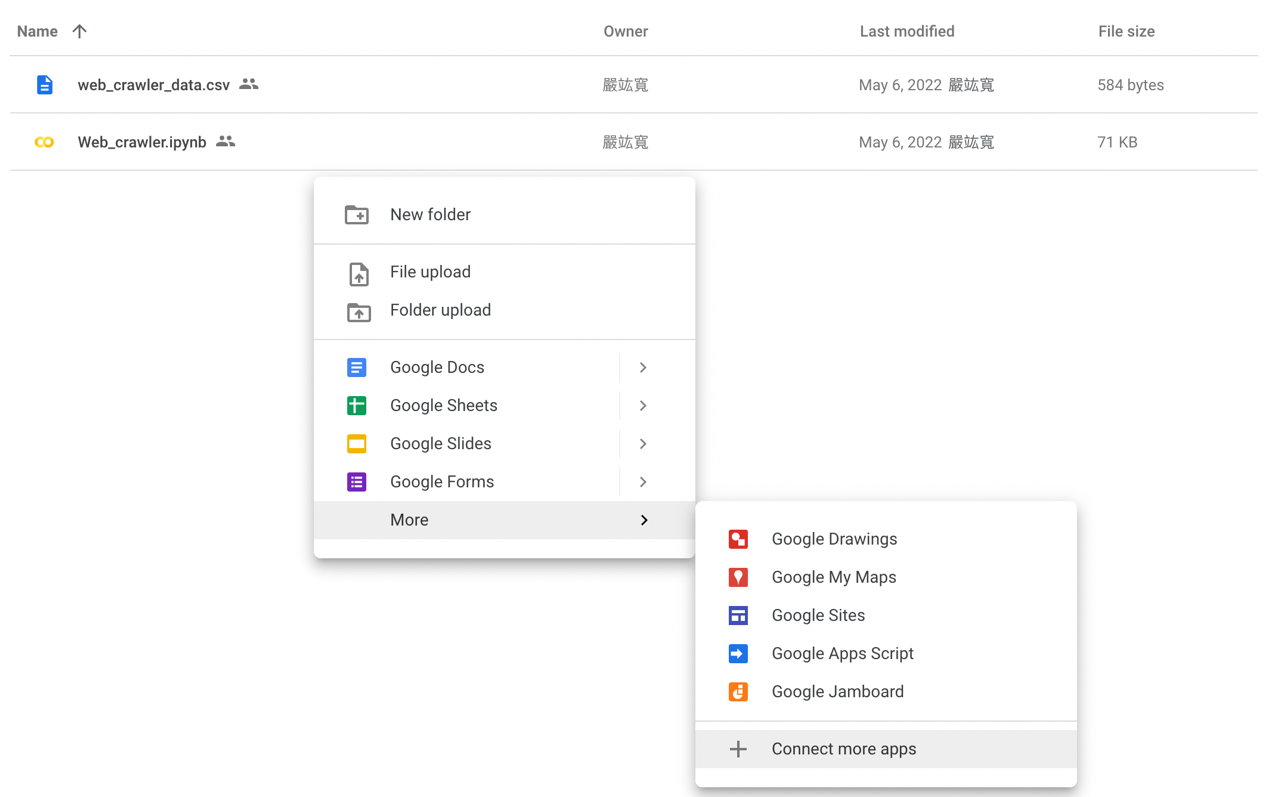

- Then you can see the application by searching “Colaboratory”.

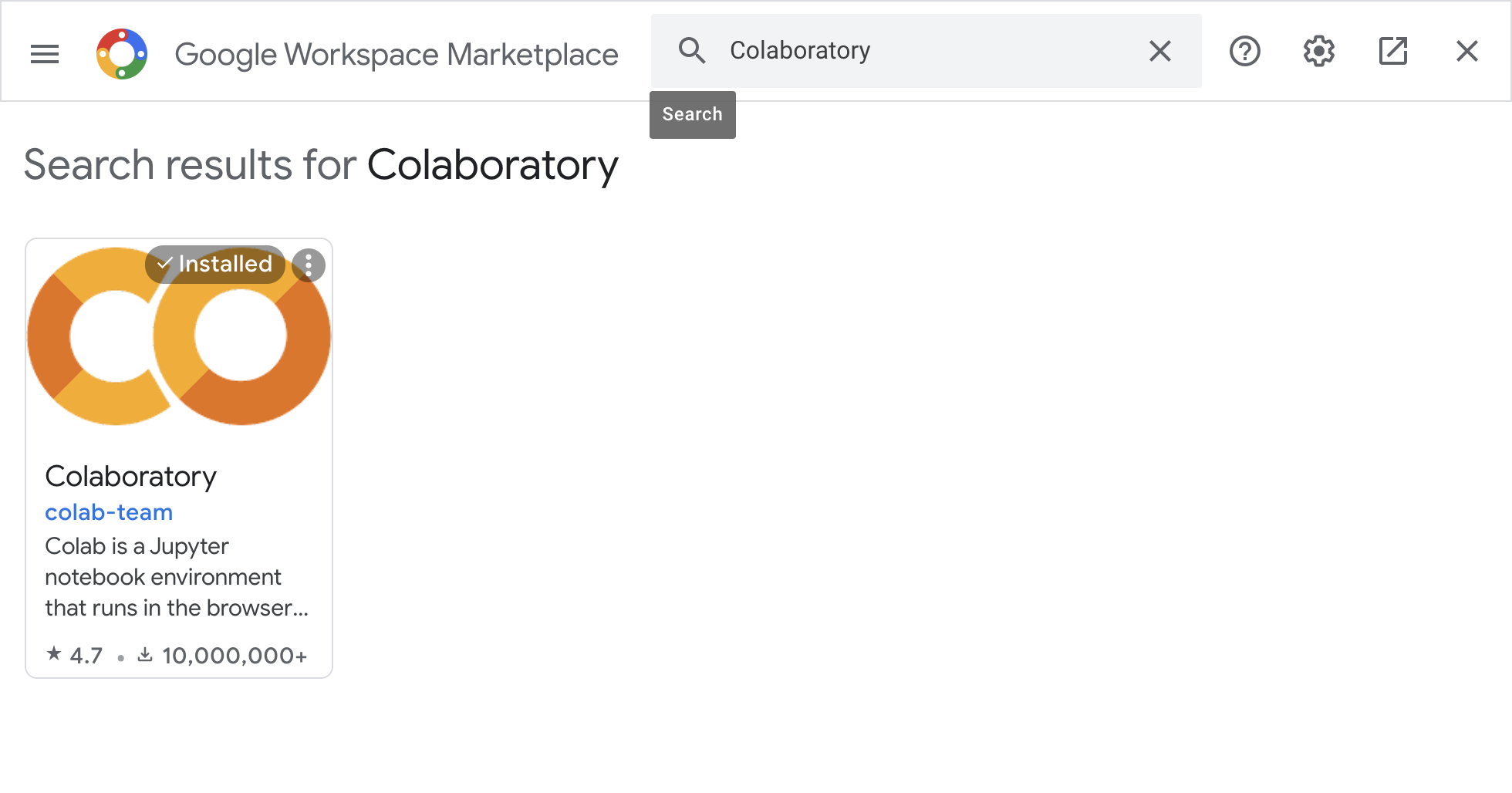


**Step 2.**

- Go to the following website, <https://drive.google.com/drive/folders/1EyswOasy8TJNGpMYWWLelcP4K9d2Vdqo?usp=sharing>
  There should be two files in the folder.
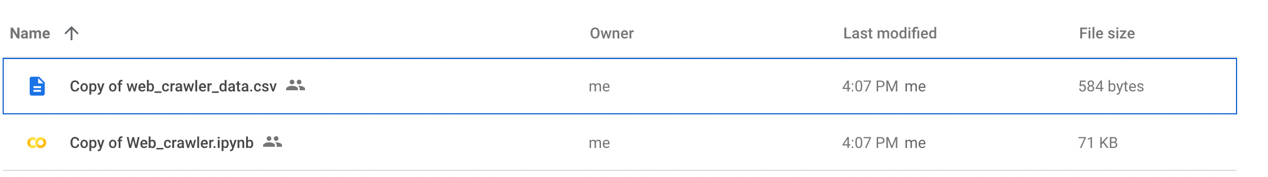

- Open the CSV file in the shared folder (the blue one).
- Choose an arbitrary cell and press ctrl + A. Then press ctrl + C.
- Then, go back to your own CSV file (the one in your Google drive), and press ctrl + V.
- Therefore, your CSV file should look exactly like this,

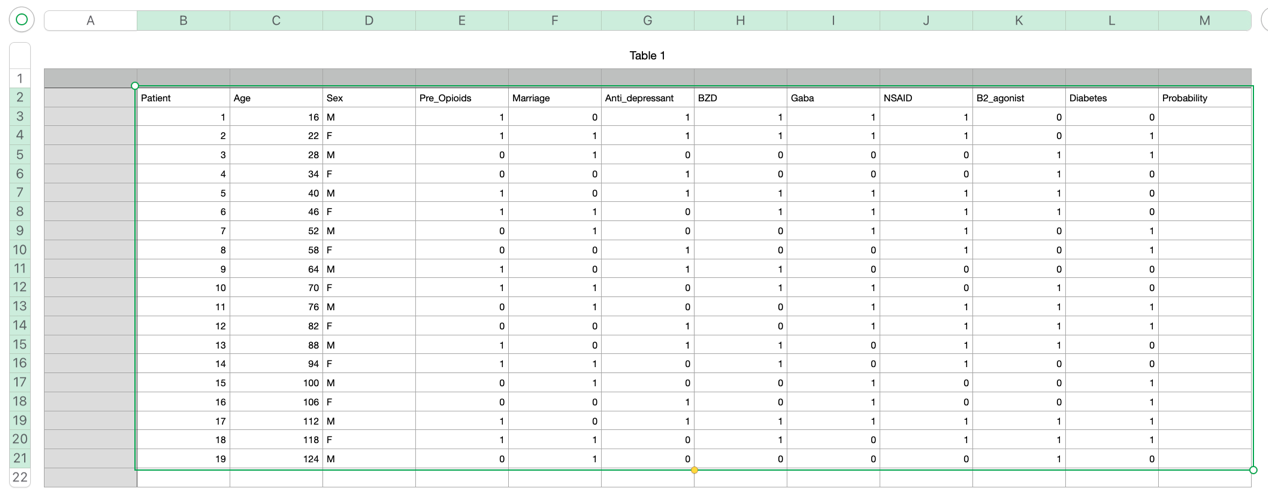

- Since this is a dummy dataset (a fake one), you should paste the data from your own cohort without changing the name of each column so you can make your own calculations.
- Notice! The coding and the order of columns should remain the same, and you should use “1” or “0” to refer patients with or without instrumentation.
- The column of “Probability” should remain blank.

**Step 3**.

- Switch to the Google Colab in the shared folder (the yellow one).
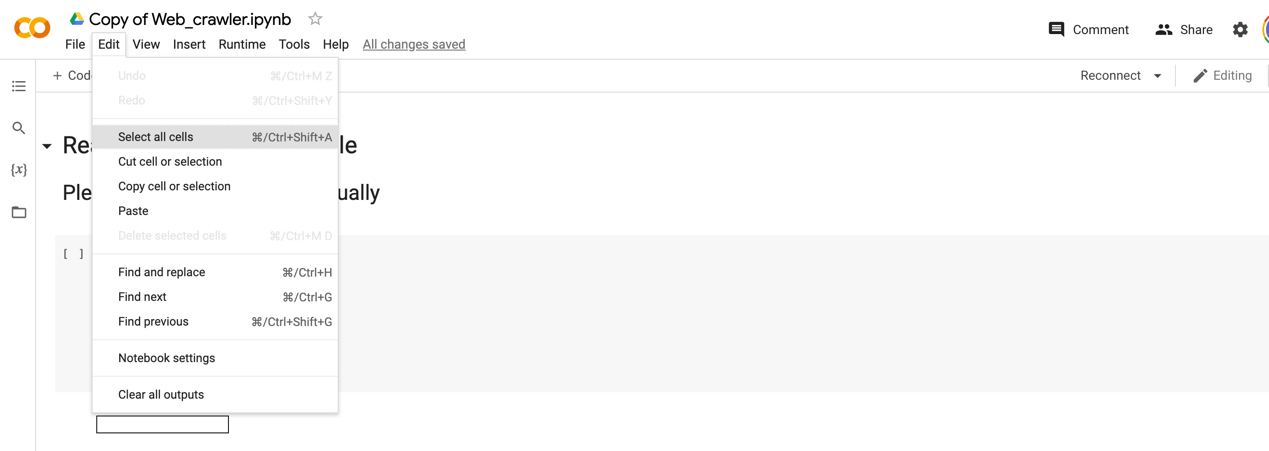

- Click “Edit”, then choose “Select all cells”, or simply press ctrl + shift + A to select the text. Then, press ctrl + C.
- Paste the code to your own colab file.

**Step 4.**

- Press right at the end of the second line in the first cell “*drive.mount ('/content/drive')*” Then, click the triangle in the circle at the top left corner (the run button).
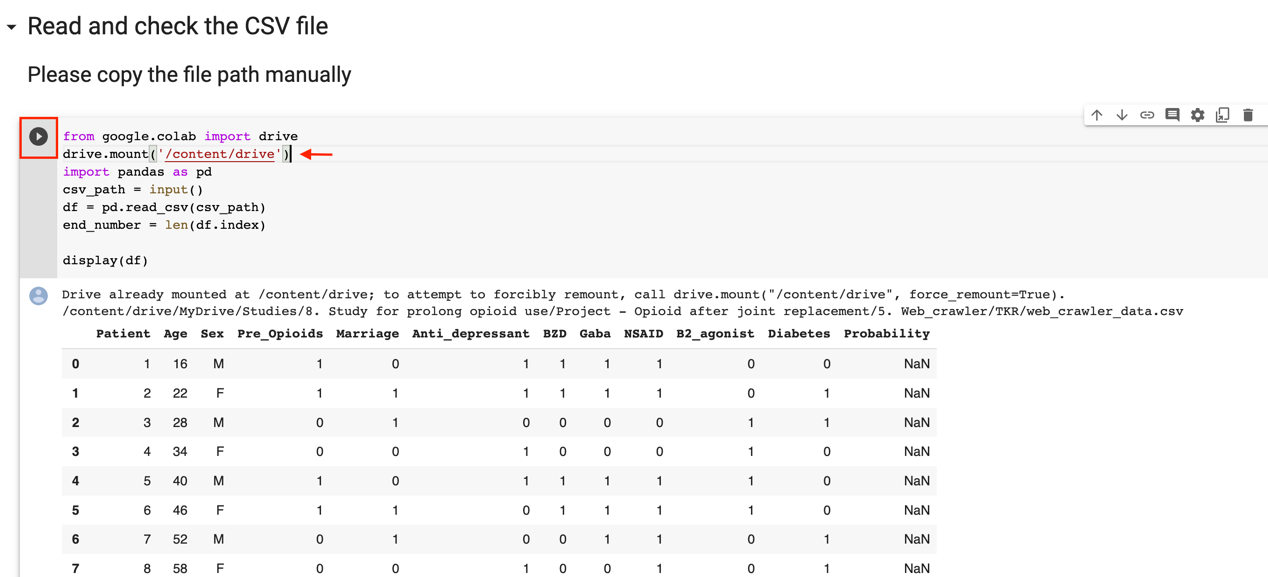

- It should give you a space to enter the path of the file. Press the file icon on the left, then find your own CSV file in your google drive. Click the button of three dots on the right, then select “Copy path”.
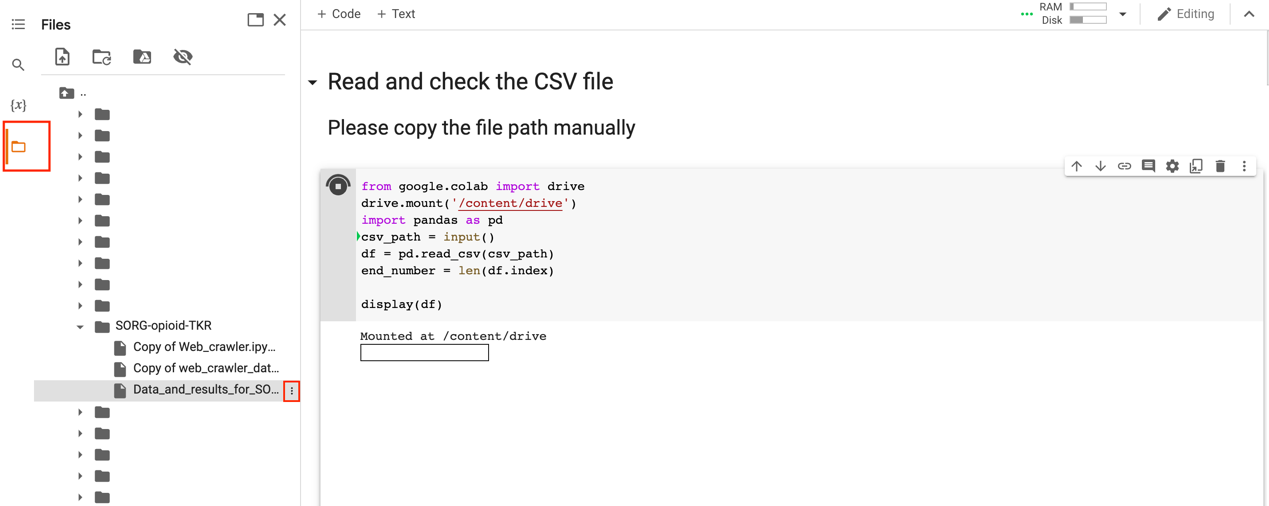

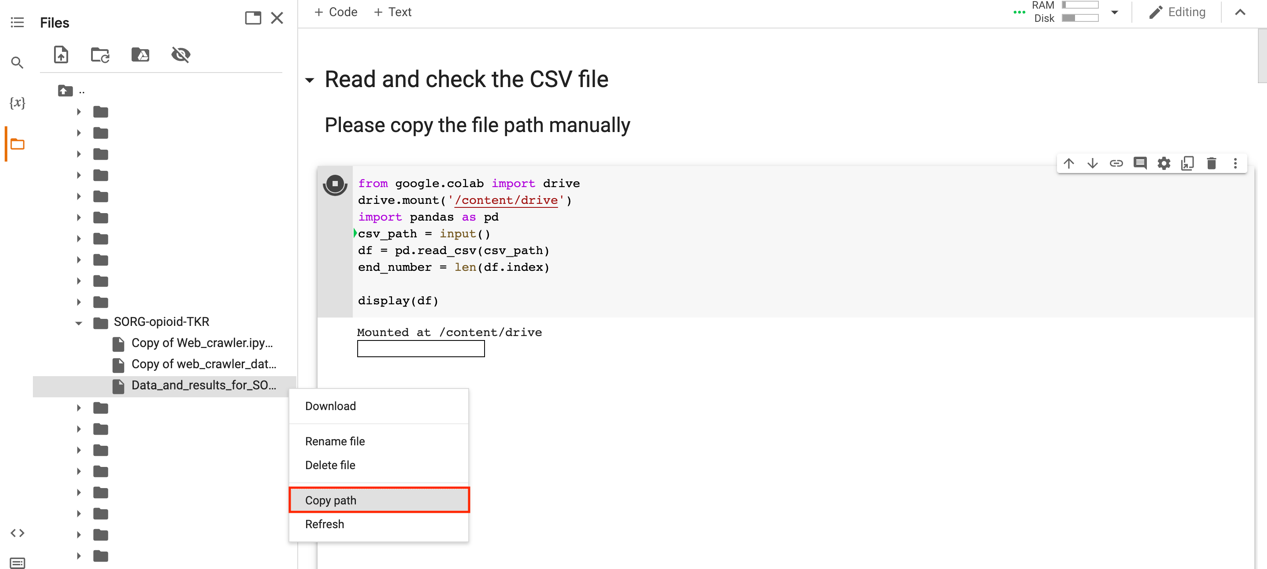

- Press ctrl + V to paste the file path in the space, then press enter.
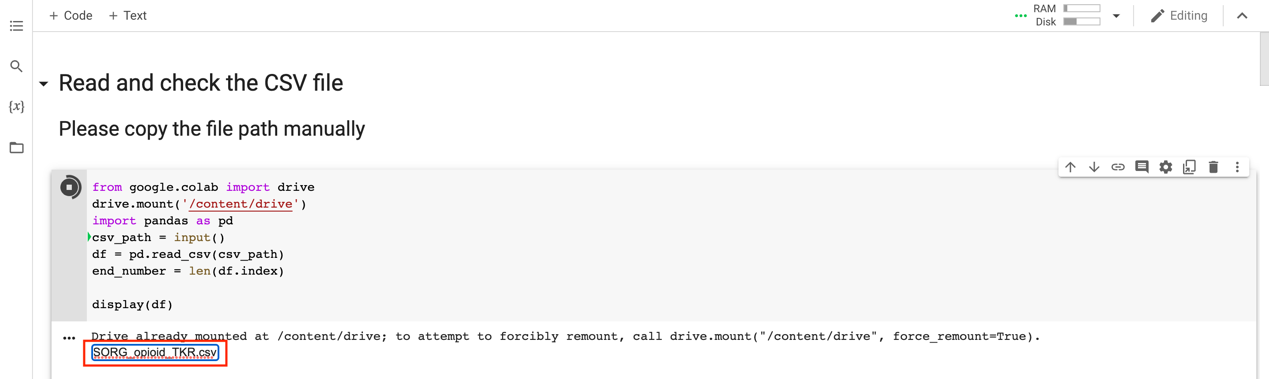


If the task was done, you should see scenes like the following screenshot.
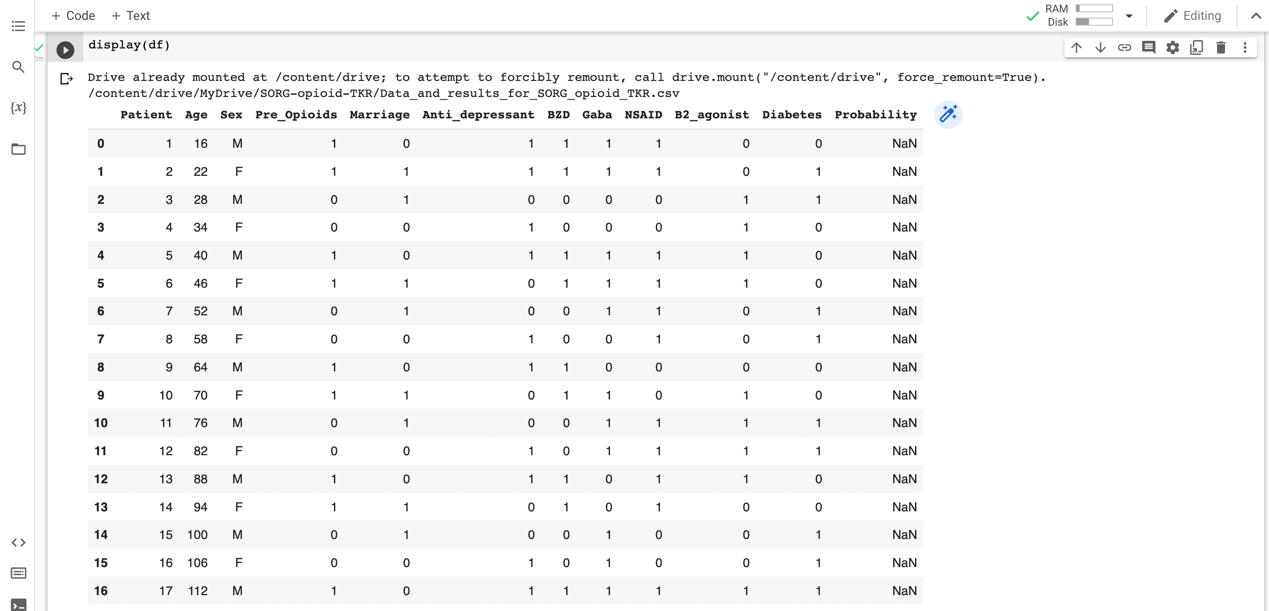


**Step 5.**

- Go to the next cell (Prepare the web crawler) and click the run button.
- Go to the next cell (Prepare necessary functions) and click the run button.
- Go to the next cell (Crawling) and click the run button in the first cell.
- It will give you a space to enter an integer right below “*Which patient do you want to start with?*”.

If you were first time using this code, you should type the number “1”, then press enter.

*
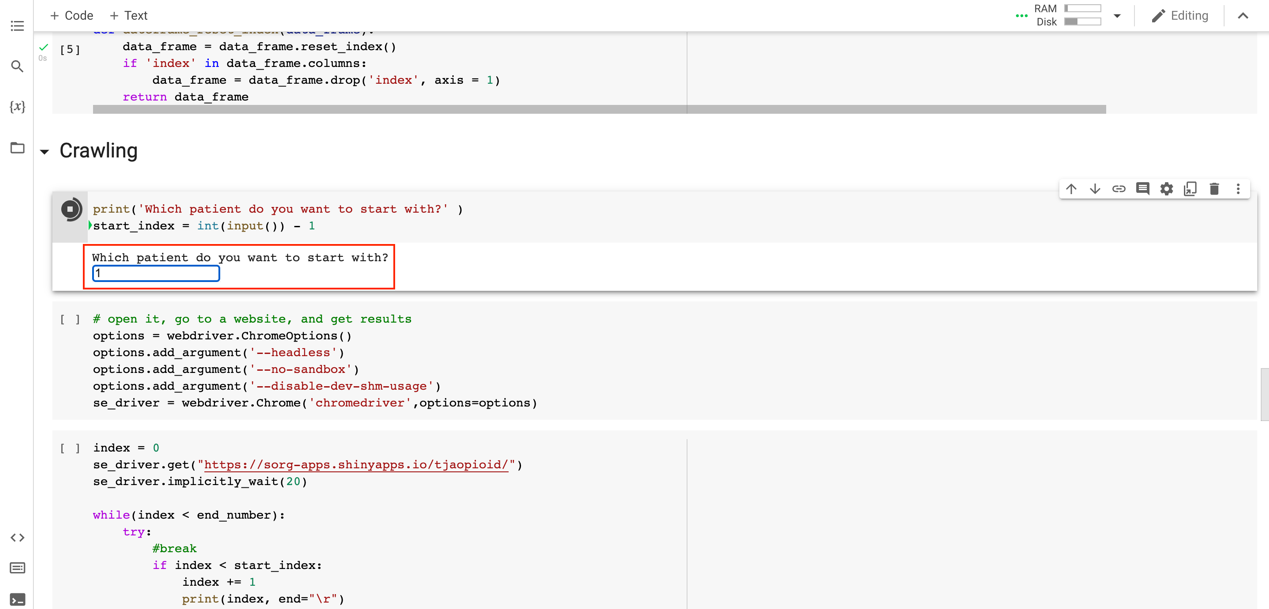
*

- Click the run button in the second and the third cell.
- Then, the machine is going to calculate the prediction probability automatically. Just give it some time, and it should print the results below. In the meantime, you could let the machine do the chores.
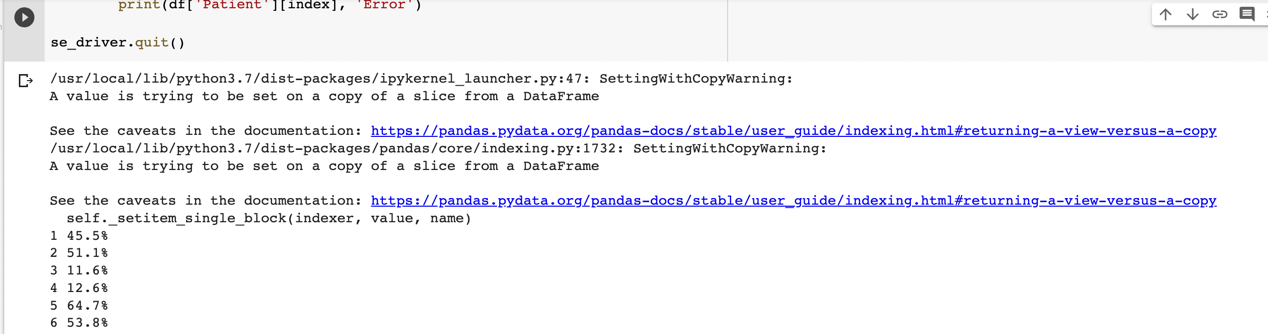

- After the task is finished, you can check your CSV file in your google drive. You should find the prediction results were already written down.
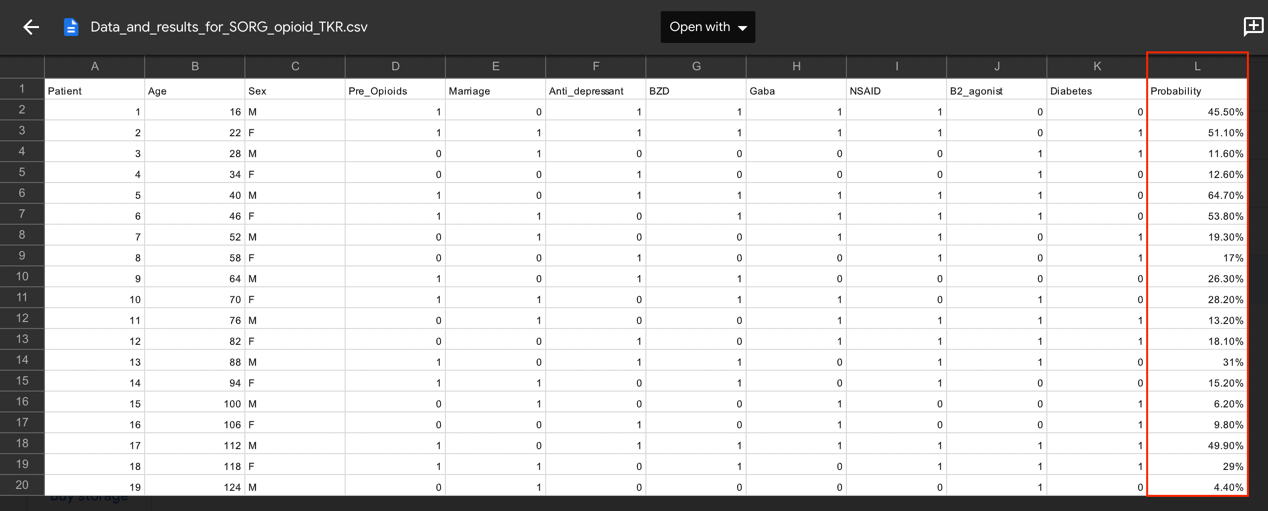


**Appendix 2**. Detailed explanation of the applied statistical methods.

Discrimination is the ability to identify high-risk groups from low-risk groups, and it is usually measured by AUROC and AUPRC. AUROC is theoretically ranged from 0.5 to 1 while the lower limit of AUPRC depends on the cohort itself. An AUROC indicates a perfect discrimination; an AUROC greater than 0.7 often indicates clinically acceptable discriminatory ability; an AUROC of 0.5 suggests the worst possible discriminatory ability. However, in low-prevalence diseases, an AUROC may give an overoptimistic view of the performance of a diagnostic test, due to the influence of false-positive cases diluted and masked by the huge number of true-negative cases. Unlike AUROC, AUPRC concerns the tradeoff between precision (instead of specificity) and recall (also known as sensitivity), and the false-positive cases were evaluated along with the true-positive cases to avoid masking and diluting effect described above. Therefore, AUPRC is believed to be more reliable when the dataset is imbalanced. Whereas, the interpretation of AUPRC results is more complicated than that of AUROC results. Unlike the theoretical lower bound of AUROC fixing at 0.5, the baseline of an AUPRC is equal to the prevalence of the event of interest. This means the benchmark AUPRCs might vary in different cohorts. A model achieves perfect AUPRC when it finds all the positive examples (perfect recall) without accidentally marking any negative examples as positive (perfect precision). The delta method was applied to calculate the 95% confidence interval (95% CI) of an AUPRC and to guarantee the interval not exceeding 1 or 0.

Calibration concerns average risk in a population. We assumed that the prediction model is well calibrated so that close to *x* of 100 subjects, with a predicted probability of *x*%, actually encountered the event of interest. A graphical assessment of calibration is the predicted probability on the x-axis, and the observed probability on the y-axis. The calibration plot can be characterized by an intercept, which indicates the extent that predictions are systematically too low or too high, and a calibration slope, which should be 1. The Brier score is a combined measure of discrimination and calibration, and was performed to indicate overall model performance. A Brier score can take on any value between 0 and 1. The lower the Brier score, the more accurate the prediction is. A null-model Brier score, which is the score for an algorithm that predicts a probability equal to the population prevalence of the outcome for every patient, should be considered as the benchmark.

DCA is a method to assess the value of information provided by a diagnostic test by considering the likely range of a patient’s risk and benefit preference, which should be quantified through a comprehensive shared decision-making process. In the decision curve, net benefit is plotted against threshold probability. The decision curve of a model is compared to extreme cases that treat all patients or none. A model is of clinical utility if the net benefit of a model is greater than treating all and none patients. It is distinguished from other statistical methods, like AUROC, by the ability to assess the clinical value of a predictor. For example, when the clinical risk is low, such as ordering antibiotics for infected patients, the clinician and the patient could choose a relatively low threshold probability. In contrast, a higher threshold probability should be applied in a scenario with low clinical benefit but high clinical risk, such as performing extensive surgery to a fragile patient. Although there is no readily available summary measure for evaluating the predictive performance between two decision curves, it is still an essential validation tool on top of measures such as discrimination and calibration.
